# “Drug resistance associated membrane proteins”

**DOI:** 10.3389/fphys.2014.00108

**Published:** 2014-03-20

**Authors:** Katy S. Sherlach, Paul D. Roepe

**Affiliations:** Department of Chemistry and Department of Biochemistry and Cellular and Molecular Biology, Georgetown UniversityWashington, DC, USA

**Keywords:** ABC transporter, *P. falciparum* chloroquine resistance transporter (PfCRT), volume regulation, active transport, drug transport

Study of drug resistance began in the late 1940's, and recognition that altered membrane transport of drug was often related to cellular drug resistance followed approximately 20 years later. Identification and isolation of specific membrane proteins that influence this altered drug transport began in the 1970's and is a major ongoing endeavor to this day. In this article, we refer to such proteins as “drug resistance associated membrane proteins,” or “DRAMPs.” In over 40 years, dozens of books and tens of thousands of research articles have asked how drug resistance is mediated by various DRAMPs. By comparison, relatively few studies have probed the normal physiological function of these proteins. In many cases, deletion of the gene encoding a DRAMP is not lethal, showing that the function of the protein is non-essential, but in some cases (e.g., PfCRT protein involved in antimalarial drug resistance) deletion is not possible, suggesting an essential function. For some DRAMPs a clear role in specific cell biological processes has been established (e.g., Ishikawa et al., 1997; Jin et al., 2002; Baugh et al., 2012; Quazi and Molday, 2013), but in most cases we are no closer to a detailed molecular definition of the physiologic function of DRAMPs than we were when these proteins were first discovered. When they are involved in drug resistance, DRAMPs are often either mutated or overexpressed, and in some cases both. Quantitative comparison between wild type and mutant isoforms of DRAMPs, or between normal and higher levels, is often quite difficult for a variety of technical reasons, and this has probably slowed elucidation of their physiologic function.

The vast majority of genetic, biochemical, biophysical, and cell biological studies with DRAMPs have emphasized their putative interactions with drugs, and dozens of review articles summarize decades of such work (e.g., Saidijam et al., 2006; Bay et al., 2008; Blair and Piddock, 2009; Damme et al., 2011). The known array of DRAMPs is now dizzying, with hundreds of proteins now organized into five families (ABC, MATE, MFS, SMR, RND), as described elsewhere (Alvarez-Ortega et al., 2013). Members of each family can be found in multiple phyla, but sequence conservation across phyla is typically quite low. Individual members of these families have been implicated in anticancer, antibacterial, antifungal, and antiparasitic drug resistance phenomena. Other papers in this volume describe specific proteins in detail. Collectively, these data related to DRAMP structure and function and their roles in drug resistance are exceedingly rich, and encompass a remarkable diversity of function. It is therefore a challenge to view them as a single class of proteins, since their only common thread is participation in drug resistance phenomena, which are biologically and chemically quite diverse.

To add additional complexity, there are four possible routes to cellular drug resistance: (1) catabolism of the drug, (2) mutation and/or altered expression of the drug target, (3) switching off a relevant metabolic pathway or (4) altered cellular transport of the drug. All operate simply to reduce the efficiency of interaction between the drug and its molecular target, and membrane proteins involved in drug resistance phenomena could in theory influence any of the four routes. To date, most DRAMPs studied in depth have been associated with altered drug transport phenomena that act to promote lower drug-drug target association. The most famous of these is human MDR1 protein (P-glycoprotein), which mediates decreased accumulation of anticancer drugs in tumor cells (Roepe et al., 1996; Quazi and Molday, 2013). Early on it was appreciated that altered drug transport induced by overexpression of huMDR1 could in theory be direct or indirect (Roepe et al., 1996), meaning huMDR1 could mediate direct translocation of drugs back out of the cell to reduce net accumulation, or indirectly influence accumulation of drugs through physical chemical effects, such as changes in membrane potential that would then effect passive influx of some drugs (Wadkins and Roepe, 1997). Decades later, multiple examples of both direct and indirect phenomena can be found for various examples of drug resistance mediated by DRAMPs. Another question raised early on was whether degrees of resistance or patterns of drug “cross resistance” were mediated solely by huMDR1 and other DRAMPs. In addressing this question, of note is the fact that many early models of drug resistant cells were created by incremental exposure to increasing concentrations of a single drug (Biedler and Riehm, 1970), protocols that induce a variety of “epi-phenomena” that are now known to add to drug resistance, along with DRAMPs. For example, it is now appreciated that the 100–1000's-fold levels of drug resistance observed in early drug selected tumor cell models are clearly not due to huMDR1 overexpression alone. A crucial concept that emerged from this period is that altered cell signaling related to programmed cell death is likely more relevant for high levels of drug resistance in many cancers, relative to the contribution made by ABC DRAMPs (Lowe et al., 1993; Borst, 2012).

A central, remaining question for most of these proteins is what functions do DRAMPs have in the absence of drugs to which resistance has been selected? Even if not essential in many cases, clearly these functions are important since the proteins are chromosomally encoded, are found in all phyla, and have been evolving for millions of years. As one example, even in the absence of man-made drug pressure for isolated microbiomes, some intrinsically drug resistant bacteria have been discovered suggesting that one normal physiologic function is conferring resistance to commensal defensins or other excreted natural antibiotics (Bhullar et al., 2012). Most drug resistance researchers believe that definition of these “normal” or “physiologic” functions will better illuminate the molecular mechanism of how the membrane proteins confer drug resistance. In examples where intrinsic drug resistance is not the physiologic function, presumably, mutations or increased expression seen for these proteins in drug selected cells confers drug resistance via the normal function being “hijacked” or altered in such a way as to reduce interaction between drug and drug target. But beyond the example of “naturally resistant” bacteria (Bhullar et al., 2012), what are those normal functions?

Other bacterial DRAMPs have been associated with a variety of natural functions that include: spermidine degradation, pH homeostasis, alkali tolerance, removal of fatty acids, bile salts, homoserine lactones, or aromatic hydrocarbons (Neyfakh, 1997; Krulwich et al., 2005; Fernándex and Hancock, 2012). For huMDR1 and its many relatives in the ABC transporter superfamily, the most illuminating early experiments came from gene knockout experiments with mice (Smit et al., 1993). Here, Borst and colleagues showed a distinct tissue/cellular phenotype associated with deletion of murine orthologs of huMDR1, namely, altered traffic of phospholipids. This suggests that the normal function of huMDR1 may include maintenance of cell membrane lipid disposition, or direct transport of phospholipids and related molecules. These hypotheses were supported in several follow-up reports (e.g., van Helvoort et al., 1996; Raggers et al., 2000; Romsicki and Sharom, 2001). Work in this area remains ongoing, and the only universally accepted conclusion at this point seems to be that there is a wider-than-expected diversity of natural substrates for ABC transporters involved in drug resistance phenomena.

Another important class of DRAMPs is comprised of those involved in anti-parasitic drug resistance. Although they are not the only mechanism that confers resistance, transporters have been implicated in drug resistance for the microorganisms that cause schistosomiasis (Kasinathan and Greenberg, 2012), leishmaniasis (Aït-Oudhia et al., 2011), sleeping sickness (Wilkinson and Kelly, 2009), malaria (Roepe, 2011), and other parasitic diseases. Perhaps the most heavily studied to data are malaria DRAMPs. These include orthologs of huMDR1 and huMRP that are found in multiple species of malarial parasites, including *P. falciparum* and *P. vivax*. Early on Wilson et al. (1989) and Foote et al. (1990), PfMDR1 was thought to be the major contributor to the most widespread form of antimalarial drug resistance [chloroquine (CQ) resistance, (CQR)], however, mutations in a novel DRAMP with no known orthologs in other eukaryotes, called PfCRT, was subsequently found to be responsible for a much larger portion of the shift in CQ cytostatic activity (CQ IC_50_) observed for CQR *P. falciparum* (Fidock et al., 2000). Multiple mutations in the *pfcrt* gene are required for CQR, and these confer patterns of amino acid substitutions in the encoded PfCRT protein. The patterns reveal the geographic origin of the CQR strain or isolate and also suggest different cross-resistance patterns to related drugs (Summers et al., 2012). There are now at least 29 distinct isoforms of PfCRT known to exist, with each isoform selected by different drug-use histories in the respective geographic origin (Baro et al., 2013). For at least a decade, it has been widely suspected that these PfCRT mutations may be all that is necessary to confer resistance to multiple quinoline antimalarial drugs, and perhaps other compounds.

Recently however, in set of observations oddly reminiscent of the now-accepted concept that huMDR1 protein overexpression is only part of the explanation for tumor multidrug resistance, we now know that PfCRT mutations are only part of the story for CQR. From cancer cells to malarial parasites, altered signaling related to drug induced programmed cell death also appears necessary for high-level drug resistance (e.g., Lowe et al., 1993; Gaviria et al., 2013). Using progeny of an available CQR × CQS parasite genetic cross, a very recent paper shows that the genetics of “cytostatic CQR” (resistance to cytostatic, or growth inhibitory, effects of the drug) are distinct from those of cytocidal CQR (resistance to cytocidal, or parasite kill, effects of the drug) (Gaviria et al., 2013). That is, similar to high levels of tumor cell multidrug resistance, and other examples of bacterial, fungal, and parasite drug resistance, it now appears likely that a DRAMP (PfCRT) is responsible for one key layer to antimalarial drug resistance, with altered signaling related to parasite cell death adding to the phenotype upon acquisition of high levels of cytocidal drug resistance (Sinai and Roepe, 2012; Gaviria et al., 2013; Roepe, 2014).

What does this tell us about studies that probe DRAMP physiologic function? An important lesson is that defining physiologic function using drug pressured or drug selected cell lines can confuse analysis due to the presence of unforeseen epi-phenomena, such as altered metabolism and signal transduction related to cell death pathways. Similar to defining the precise contribution of the DRAMP to drug resistance, it can be difficult in these drug selected systems to unambiguously define physiologic function because other epi-phenomena are present. Direct gene knockout experiments can in some cases be informative, except of course in examples where the DRAMP is essential for cell viability (Waller et al., 2003). The ultimate goal for molecular definition of DRAMP function is purification and reconstitution into a lipid bilayer or proteoliposome preparation, followed by direct molecular assays. A number of such experiments have been done for huMDR1 and other eukaryotic ABC transporters (e.g., Sharom et al., 1993; Ambudkar et al., 1998; Howard and Roepe, 2003), bacterial DRAMPs (e.g., Yerushalmi et al., 1995), and even malarial DRAMPs (Paguio et al., 2009). Some of these studies have been used to query possible natural substrates, but many more such experiments are needed.

Even once the protein is purified and reconstituted, functional assays for DRAMPs can be quite difficult, in part because the suspected transporter substrates are often hydrophobic molecules or lipophilic metabolites. Also, some DRAMPs may not even be transporters, but signaling receptors involved in cell death that require yet unknown co-factors. In the case of hydrophobic substrates, routine transport assays that separate proteoliposomes from substrate at various time points via centrifugation or filtration have very high background due to the substrate adhering to lipid, filters, etc. Another approach that shows promise, at least for known substrates, is to use a fluorometric substrate analog wherein a fluorescent signal can be used to distinguish drug on one side of a membrane vs another. For example, this has recently been used to monitor CQ transport by PfCRT (Paguio et al., 2009). This analysis reveals features of drug transport by PfCRT that are not possible to monitor with other approaches (Roepe, 2011).

Yet, similar to most other DRAMPs, for PfCRT, and PfMDR1, we still have no unequivocal molecularly defined function for the protein in normal cell physiology. For PfCRT cell biological clues abound, and in particular include some evidence for a role in organellar volume regulation, ionic equilibria, hemoglobin metabolism, and perhaps glutathione traffic (Bennett et al., 2004; Gligorijevic et al., 2006; Patzewitz et al., 2013; Lewis et al., 2014). Further testing these hypotheses, with purified protein in reconstituted systems such as proteoliposomes and bilayers, remains an important goal as is the case for all DRAMPs. The papers in this volume offer invaluable guidance in this ongoing endeavor.
